# Increase in Serum Soluble Tim-3 Level Is Related to the Progression of Diseases After Hepatitis Virus Infection

**DOI:** 10.3389/fmed.2022.880909

**Published:** 2022-05-12

**Authors:** Lingli Chen, Xiaomei Yu, Chunyan Lv, Yaping Dai, Tao Wang, Shaoxiong Zheng, Yuan Qin, Xiumei Zhou, Yigang Wang, Hao Pei, Hongming Fang, Biao Huang

**Affiliations:** ^1^College of Life Sciences and Medicine, Zhejiang Sci-Tech University, Hangzhou, China; ^2^Wuxi No.5 People’s Hospital, Wuxi, China; ^3^Affiliated Xiaoshan Hospital, Hangzhou Normal University, Hangzhou, China

**Keywords:** viral hepatitis, liver fibrosis, soluble Tim-3, time-resolved fluorescence immunoassay, diagnostic value

## Abstract

**Background:**

Viral hepatitis is a widespread and serious infectious disease, and most patients with liver cirrhosis and hepatocellular carcinoma are prone to viral infections. T cell immunoglobulin-and mucin-domain-containing molecule-3 (Tim-3) is an immune checkpoint molecule that negatively regulates T cell responses, playing an extremely important role in controlling infectious diseases. However, reports about the role of serum soluble Tim-3 (sTim-3) in hepatitis virus infection are limited. Therefore, this study explored changes in sTim-3 levels in patients infected with hepatitis B virus (HBV), hepatitis C virus (HCV), and hepatitis E virus (HEV).

**Methods:**

This study applied high-sensitivity time-resolved fluorescence immunoassay for the detection of sTim-3 levels. A total of 205 cases of viral hepatitis infection (68 cases of HBV infection, 60 cases of HCV infection, and 77 cases of HEV virus infection) and 88 healthy controls were quantitatively determined. The changes in serum sTim-3 level and its clinical value in hepatitis virus infection were analyzed.

**Results:**

Patients with HBV infection (14.00, 10.78–20.45 ng/mL), HCV infection (15.99, 11.83–27.00 ng/mL), or HEV infection (19.09, 10.85–33.93 ng/mL) had significantly higher sTim-3 levels than that in the healthy control group (7.69, 6.14–10.22 ng/mL, *P* < 0.0001). Patients with hepatitis and fibrosis infected with HBV (22.76, 12.82–37.53 ng/mL), HCV (33.06, 16.36–39.30 ng/mL), and HEV (28.90, 17.95–35.94 ng/mL) had significantly higher sTim-3 levels than patients with hepatitis without fibrosis (13.29, 7.75–17.28; 13.86, 11.48–18.64; 14.77, 9.79–29.79 ng/mL; *P* < 0.05).

**Conclusion:**

sTim-3 level was elevated in patients infected with HBV, HCV, or HEV and gradually increased in patients with either hepatitis or hepatitis with hepatic fibrosis. It has a certain role in the evaluation of the course of a disease after hepatitis virus infection.

## Introduction

Hepatitis virus infection is one of the major public health concerns globally with high mortality and morbidity, affecting hundreds of millions of people (1). In addition to hepatitis A virus (HAV), hepatitis B virus (HBV), hepatitis C virus (HCV), hepatitis D virus (HDV), and hepatitis E virus (HEV) cause chronic infections. HBV and HCV are the main causes of chronic diseases and liver cancer (2). Viral hepatitis is caused by the complex interaction between the replication of the hepatitis virus and the host immune response (3). CD8 + T cells play an important role in immune response to viral infections. In acute hepatitis virus infection, CD8 + T cells mainly eliminate the virus by producing tumor necrosis factor-α (TNF-α) and interferon-γ (IFN-γ) (4). In chronic hepatitis infection, as infection progresses, the effector functions of T cells gradually decrease (including the reduction of proliferation and cytotoxicity), and their ability to produce IL-2, TNF-α, and IFN-γ decreases (5, 6). That is, CD8 + T cells show a depleted phenotype. The degree of depletion of hepatitis virus-specific CD8 + T cells showcase varying clinical results (7–10).

T cell immunoglobulin-and mucin-domain-containing molecule-3 (Tim-3) is a type I transmembrane protein that negatively regulates immune response and is involved in the dysfunction and failure of CD8 + T cells in HBV and HCV infections (11). Tim-3 expression increases in HBV/HCV-specific CD8 + T cells, and this increase is related to the failure of CD8 + T cells in patients with HBV/HCV infection (12–15). In addition, the combination of Tim-3 and its ligand galectin-9 can increase the expression level of the Tim-3 protein during the activation of regulatory T (Treg) cells, and the increase in Tim-3 level can induce helper T lymphocyte 1 (Th1) cell apoptosis and inhibit Th1-type immune response, thereby regulating Th1/Th2 balance in patients with hepatitis B (13, 16). The immune function of membrane-bound Tim-3 during chronic HBV and chronic HCV infection and its role in diseases has been explored, but the clinical importance of changes in serum soluble Tim3 (sTim-3) levels during viral hepatitis, especially HEV infection, remains unclear. Therefore, this study detected the level of sTim-3 in the serum of patients with different viral hepatitis infections (HBV, HCV, and HEV), and analyzed the diagnostic value of serum sTim-3 in these patients.

## Patients and Methods

### Serum Samples

A total of 205 patients with hepatitis virus infection from Zhejiang Xiaoshan Hospital and Wuxi NO.5 People’s Hospital were included in the study. Patients comprising 68 patients infected with HBV (46 patients with hepatitis and 22 patients with hepatitis and fibrosis), 60 patients infected with HCV (47 patients with hepatitis and 13 patients with hepatitis and fibrosis), and 77 patients infected with HCV (50 patients with hepatitis and 27 patients with hepatitis and fibrosis). In addition, 88 healthy individuals were also included in the study. The samples were included in two batches successively. The first batch included 50 healthy people, 90 hepatitis patients, and 32 patients with hepatitis and fibrosis. The first batch was included from 2020.1 to 2020.6. The second batch was included 38 healthy people, 53 hepatitis patients, and 30 patients with hepatitis and fibrosis. The second batch was included from 2020.7 to 2020.12. The healthy control group was composed of adults who came to the hospital for physical examination and had no history of liver disease and were negative for HBV, HCV, and HEV infections. A total of 68 patients tested positive for hepatitis B surface antigen positivity, 60 patients tested positive for anti-HCV IgG positivity, and 77 patients tested positive for anti-HEV IgG positivity. Patients with hepatitis and fibrosis had undergone liver biopsy with significant fibrosis (METAVIR status F3-F4; F3, numerous septa without cirrhosis; and F4, cirrhosis). A complete medical history was taken and physical examination carried out in all patients and controls. Exclusion criteria were HIV infection, HAV infection, auto-immune hepatitis, non-alcoholic fatty liver diseases (excluded based on histopathological findings), patients with uncontrolled psychiatric disorders, cardiac diseases (cardiomyopathy, arrhythmias, ischemia, myocarditis and significant valvular disease), severe comorbid diseases (renal failure, hypertension), congenital liver disease, a history of alcohol intake and pregnancy.

Serum collection and storage: venous blood (5 mL) was collected from each participant and centrifuged at 4,000 rpm for 10 min. The supernatant (serum) was stored at −80^°^C for subsequent use. Serum alanine aminotransferase (ALT), aspartate aminotransferase (AST), albumin (ALB), hyaluronic acid (HA), direct bilirubin (Dbil), hepatitis B surface antigen (HBsAg), and total bilirubin (Tbil) were provided by the hospital.

The study protocol conformed to the ethical guidelines of the 1975 Declaration of Helsinki. Written informed consent was obtained from all patients and the study was approved by the hospital’s ethics committee (approval no. 2020-023-1).

### Detection Methods

Reagents and instruments: two monoclonal antibodies against different Tim-3 epitopes (capture and detection antibody), and Tim-3 antigen were purchased from Sino Biological Inc. (Beijing, China). The enhancement solution was provided by Zhejiang Boshi Biotechnology Co., Ltd., ProClin-300, Tris, Sephadex-G50, bovine serum albumin (BSA), and other reagents were purchased from Shanghai Xibao Biotechnology Company (Beijing, China).

A time-resolved immunofluorescence analyzer was purchased from Foshan Daan Medical Equipment Co., Ltd., and 96-well plates were purchased from Xiamen Yunpeng Technology Development Co., Ltd.

Buffer compositions: coating buffer (50 mmol/L Na_2_CO_3_-NaHCO_3_; pH 9.6); elution buffer (50 mmol/L Tris-HCl, 0.2% BSA, and 0.05% ProClin; pH 7.8); washing buffer (50 mmol/L Tris-HCl, 0.9% NaCl, 0.02% Tween-20, and 0.01% Proclin300; pH 7.8); blocking solution (50 mmol/L Tris-HCl, 0.9% NaCl, 1% BSA, and 0.05% NaN_3_; pH 7.8); labeling buffer (50 mmol/L Na_2_CO_3_-NaHCO_3_; pH 9.0); analysis buffer (50 mmol/L Tris-HCl, 0.9% NaCl, 0.5% BSA, 0.0008% DTPA, 0.0005% PHloxine B, 0.01% Tween-40, and 0.05% Proclin300; pH 7.8).

TRFIA’s double antibody sandwich method for sTim-3 detection. The experimental steps were as follows (17):

• (1) Antibody coating: the capture antibody was diluted to 2 μg/mL with coating buffer, and 100 μL of the diluted capture antibody solution was added to each well of the 96-well plate and overnight at 4°C. The plate was washed once with washing buffer, and 150 μL of blocking solution was added to each well. After blocking at room temperature for 2 h, the blocking solution was discarded. After drying, stored at −20^°^C until further use.
• (2) Labeling antibody: 300 μg of the detection antibody was added to an ultrafiltration tube. Through ultrafiltration, the buffer of the antibody to be detected was converted into a labeling buffer with pH 9.0. The collected antibody was mixed with 30 μL of 2 mg/mL diethylenetriaminetriacetic acid (DTTA)-Eu^3+^, and the mixture was incubated at 30^°^C overnight. The next day, the labeled antibody was purified with Sephadex G50 and elution buffer. Finally, the Eu^3+^-labeled antibody (Eu^3+^-McAb) was collected and stored at −20^°^C.
• (3) The sTim-3 antigen was diluted with analysis buffer to different concentrations (6.25, 12.5, 25, 50, and 100 ng/mL), and a concentration of 0 indicated the standard buffer.
• (4) Determination of sTim-3 concentration in serum: 100 μL of standard solution or serum sample were added to a 96-well microtiter plate coated with anti-Tim-3 capture antibody. The plate was incubated at 37^°^C with shaking for 1 h and washed twice with washing buffer. Then, 100 μL of Eu^3+^-McAb (diluted 1:1000 with assay buffer) was added to each well, incubated at 37^°^C for 1 h, and washed six times with washing buffer. Approximately 100 μL of enhancement solution was added to each well, and the plate was incubated with shaking for 3 min. Finally, a time-resolved immunofluorescence analyzer was used to measure the fluorescence counts.

### Statistical Analysis

Data were expressed as median or quartile. Statistical analysis was performed using SPSS software version 21.0 (IBM Corporation, Armonk, NY, United States). Mann–Whitney test was performed for the comparison of serum indicator levels in the patients v/s controls. Binary regression was used to analyze influencing factors. The correlations among the values were determined by calculating Spearman’s correlation coefficient. GraphPad Prism (GraphPad Software Company) was used in drawing the receiver operating characteristic (ROC) curve for the determination of the best cutoff value of sTim-3 level and evaluation of the sTim-3 performance in distinguishing between the hepatitis and hepatitis with fibrosis groups.

## Results

### Association of Serum Soluble Tim-3 Levels in Hepatitis B Virus, Hepatitis C Virus, and Hepatitis E Virus Infection-Associated Disease Conditions

**FIGURE 1:**
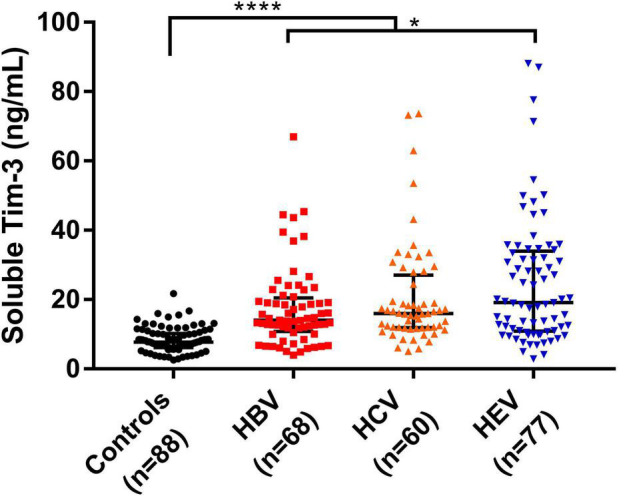
sTim-3 content in uninfected v/s in patients infected with HBV, HCV, and HEV. *P < 0.05; ****P < 0.0001.

Figure 1 shows that the sTim-3 concentrations in patients infected with HBV (14.00, 10.78–20.45 ng/mL), HCV (15.99, 11.83–27.00 ng/mL), or HEV (19.09, 10.85–33.93 ng/mL) were significantly higher than those in the healthy controls (7.69, 6.14–10.22 ng/mL, *P* < 0.0001). The sTim-3 concentrations in the HEV-infected patients were significantly higher than those in the HBV-infected patients (*P* < 0.01).

### Logistic Analysis of Factors Influencing Hepatitis or Liver Fibrosis After Hepatitis B Virus, Hepatitis C Virus, and Hepatitis E Virus Infection

Table 1 showing occurrence of hepatitis with or without fibrosis as the outcome variable, with gender, age, ALT, AST, ALB, Dbil, and sTim-3 levels as independent variables for logistic regression analysis. ALT (OR = 1.060, *P* < 0.0001; 1.047, *P* = 0.001; 1.052, *P* < 0.0001), AST (OR = 1.153, *P* < 0.0001; 1.138, *P* < 0.0001; 1.142, *P* < 0.0001), ALB (OR = 1.468, *P* < 0.0001; 1.400, *P* < 0.0001; 1.397, *P* < 0.0001), Dbil (OR = 4.274, *P* < 0.0001; 2.939, *P* < 0.0001; 2.528, *P* < 0.0001) and sTim-3 (OR = 1.332, *P* < 0.0001; 1.416, *P* < 0.0001; 1.386, *P* < 0.0001) were independent risk factors for hepatitis or liver fibrosis after HBV, HCV, and HEV infection, respectively.

**TABLE 1:** Logistic analysis of factors influencing hepatitis or liver fibrosis after HBV, HCV and HEV infection.

	Controls vs. HBV						Controls vs. HCV						Controls vs. HEV					
	β	*S.E*	*Wald*	*P*	*OR*	*95%CI*	β	*S.E*	*Wald*	*P*	*OR*	*95%CI*	β	*S.E*	*Wald*	*P*	*OR*	*95%CI*
Gender	−2.092	0.429	23.813	<0.0001	0.123	0.053–0.286	−0.695	0.347	4.003	0.045	0.499	0.253–0.986	−0.018	0.391	0.216	0.642	0.834	0.387–1.795
Age	−0.032	0.011	8.064	0.005	0.948	0.948–0.990	0.001	0.012	0.012	0.914	1.001	0.978–1.025	0.024	0.014	2.926	0.087	1.024	0.997–1.052
ALT	0.058	0.012	21.794	<0.0001	1.06	1.034–1.086	0.046	0.011	16.818	0.001	1.047	1.024–1.071	0.051	0.012	17.867	<0.0001	1.052	1.028–1.077
AST	0.142	0.028	25.041	<0.0001	1.153	1.090–1.219	0.129	0.027	22.124	<0.0001	1.138	1.078–1.201	0.133	0.031	18.421	<0.0001	1.142	1.075–1.214
ALB	0.384	0.075	26.359	<0.0001	1.468	1.268–1.700	0.337	0.065	26.957	<0.0001	1.4	1.233–1.590	0.334	0.063	28.176	<0.0001	1.397	1.235–1.580
Dbil	1.453	0.258	31.6	<0.0001	4.274	2.576–7.093	1.078	0.191	31.747	<0.0001	2.939	2.020–4.277	0.928	0.212	19.132	<0.0001	2.528	1.668–3.831
sTim-3	0.287	0.051	31.274	<0.0001	1.332	1.205–1.473	0.348	0.062	31.627	<0.0001	1.416	1.254–1.598	0.326	0.069	22.449	<0.0001	1.386	1.211–1.586

*HBV, hepatitis B virus; HCV, hepatitis C virus; HEV, hepatitis E virus.*

### Correlations of Serum Soluble Tim-3 Levels With Other Parameters

**FIGURE 2:**
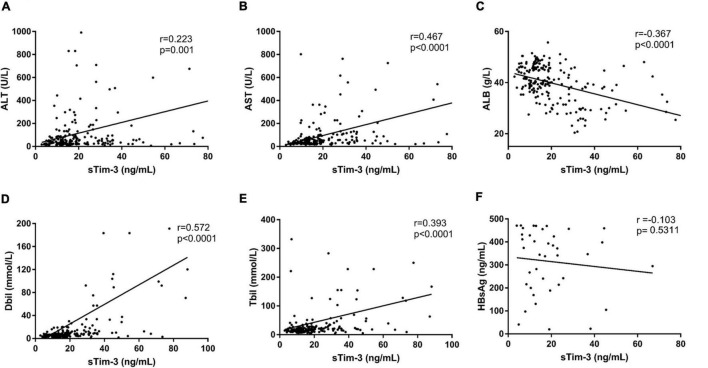
Relationship between sTim-3 levels and liver function indexes in patients with hepatitis virus infection. **(A)** Correlation analysis of sTim-3 and ALT levels showed a significant positive correlation. **(B)** Correlation analysis of sTim-3 and AST levels showed a significant positive correlation. **(C)** Correlation analysis of sTim-3 and ALB levels showed a significant negative correlation. **(D)** Correlation analysis of sTim-3 and Dbil levels showed a significant positive correlation. **(E)** Correlation analysis of sTim-3 and Tbil levels showed a significant positive correlation. **(F)** Correlation analysis of sTim-3 and HbsAg levels was not significant.

As shown in Figure 2, the sTim-3 levels in patients with HBV, HCV, or HEV infection were highly positively correlated to serum ALT (*r* = 0.223, *P* = 0.001), AST (*r* = 0.467, *P* < 0.0001), Dbli (*r* = 0.572, *P* < 0.0001), and Tbil (*r* = 0.393, *P* < 0.0001) and significantly negatively correlated with serum ALB (*r* = −0.367, *P* < 0.0001). Next, we checked for any possible correlation between HBsAg and sTim-3 levels in HBV infection. As shown in Figure 2F, we analyzed the correlation between the levels of hepatitis B virus surface antigen and sTim-3 in patients with hepatitis B virus infection; the result showed that the correlation between HBsAg and sTim-3 was not significant (*r* = -0.103, *P* = 0.5311).

### Correlation Between Serum Soluble Tim-3 Level and Hepatitis or Liver Fibrosis Caused by Hepatitis B Virus, Hepatitis C Virus, and Hepatitis E Virus Infection

**FIGURE 3:**
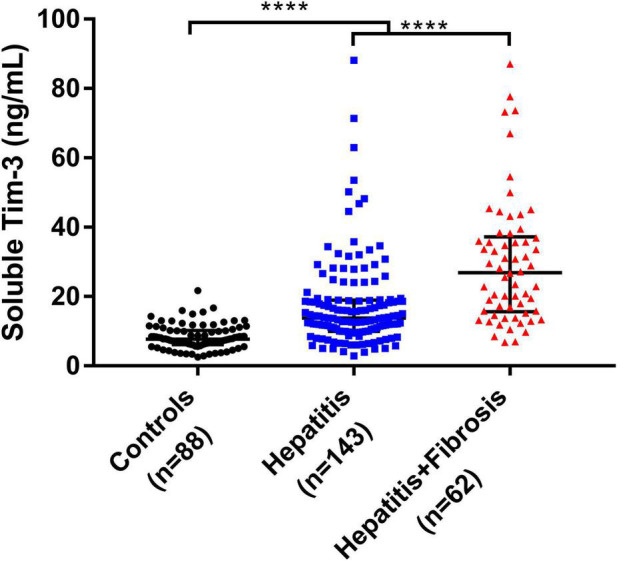
Levels of sTim-3 in normal individuals, patients with hepatitis, and patients with hepatitis and fibrosis. ****P < 0.0001.

We further divided the patients infected with HBV, HCV, or HEV into hepatitis group and hepatitis with fibrosis group. Figure 3 shows that the sTim-3 concentrations in the control, hepatitis, and hepatitis with fibrosis groups gradually increased, and the differences were significant (*P* < 0.0001).

As shown in Table 2, the sTim-3 concentrations (22.76, 12.82–37.53; 33.06, 16.36–39.30; 28.90, 17.95–35.94 ng/mL) in patients infected with HBV, HCV, or HEV with liver fibrosis were significantly higher than those in patients with hepatitis (13.29, 7.75–17.28; 13.86, 11.48–18.64; 14.77, 9.79–29.79 ng/mL). In addition, in the HBV-infected group, the serum AST, Dbil, HA, and ALB concentrations in patients with hepatitis and fibrosis were significantly different compared to those in patients with hepatitis (*P* < 0.05). In the HCV-infected group, the serum levels of ALB, Dbil, HA and Tbil in patients with liver fibrosis were significantly different from those in patients with hepatitis (*P* < 0.05). However, in the HEV-infected group, except for sTim-3 and HA, no significant differences were observed in these indicators between hepatitis and hepatitis with fibrosis groups.

**TABLE 2:** Laboratory parameters of patients with hepatitis and hepatitis with liver fibrosis caused by HBV, HCV, and HEV infections.

Variables	Controls	HBV		HCV		HEV	
		Hepatitis	Hepatitis + fibrosis	Hepatitis	Hepatitis + Fibrosis	Hepatitis	Hepatitis + fibrosis
Gender M/F	41/47	31/15	7/15	28/19	8/5	31/19	9/18
Age	56.00 (38.50–66.75)	41.50 (32.75–51.50)	55.00 (40.00–69.00)	53.00 (46.00–58.00)	55.00 (46.00–71.00)	53.00 (48.75–67.00)	59.00 (51.00–68.00)
ALT (U/L)	19.95 (16.85–25.5)	45.50 (25.00–86.00)	42.00 (28.00–241.50)	38.00 (21.00–68.00)	39.00 (21.00–81.00)	46.00 (21.00–75.00)	77.00 (32.25–233.50)
AST (U/L)	21.00 (18.80–25.97)	33.50 (25.75–57.75)	51.00 (32.00–244.50)*	36.00 (25.00–55.00)	37.00 (24.00–97.50)	55. 50 (28.00–131.25)	55.00 (33.00–108.00)
ALB (g/L)	15.20 (11.90–19.30)	46.55 (39.85–48.67)	36.10 (31.55–40.75)*	45.50 (41.70–46.40)	31.30 (29.50–39.00)*	38.90 (36.85–42.20)	31.40 (28.10–37.60)
Dbil (mmol/L)	2.70 (2.23–3.37)	5.25 (4.07–7.40)	11.50 (5.05–106.75)*	6.00 (3.90–7.37)	14.20 (8.55–33.15)*	6.20 (3.40–12.20)	14.70 (8.20–57.30)
Tbil (mmol/L)	15.20 (11.90–19.20)	15.00 (12.33–23.50)	19.00 (12.50–24.50)	17.00 (12.00–22.00)	34.00 (18.50–63.00)*	16.00 (10.87–27.75)	34.00 (19.00–63.00)
HA (ng/mL)	57 (45.00–84.00)	68.46 (37.46–102.25)	87.00 (67.00–231.79)*	81.50 (40.85–122.23)	109.00 (99.00–727.27)*	79.00 (71.00–98.50)	120.17 (92.00–503.65)*
sTim-3 (ng/mL)	7.69 (6.74–10.22)	13.29 (7.75–17.28)	22.76 (12.82–37.53)*	13.86 (11.48–18.64)	33.06 (16.36–39.30)*	14.77 (9.79–29.79)	28.90 (17.95–35.94)*

ALT, alanine aminotransferase; AST, aspartate aminotransferase; ALB, albumin; Dbil, direct bilirubin; Tbil, total bilirubin; HA, hyaluronic acid; HBV, hepatitis B virus; HCV, hepatitis C virus; HEV, hepatitis E virus.*Significant differences in hepatitis and liver fibrosis in each parameter in the same virus infection (P < 0.05).

### Diagnostic Value of Serum Soluble Tim-3 in Distinguishing Hepatitis From Hepatitis With Fibrosis After Hepatitis Virus Infection

**FIGURE 4:**
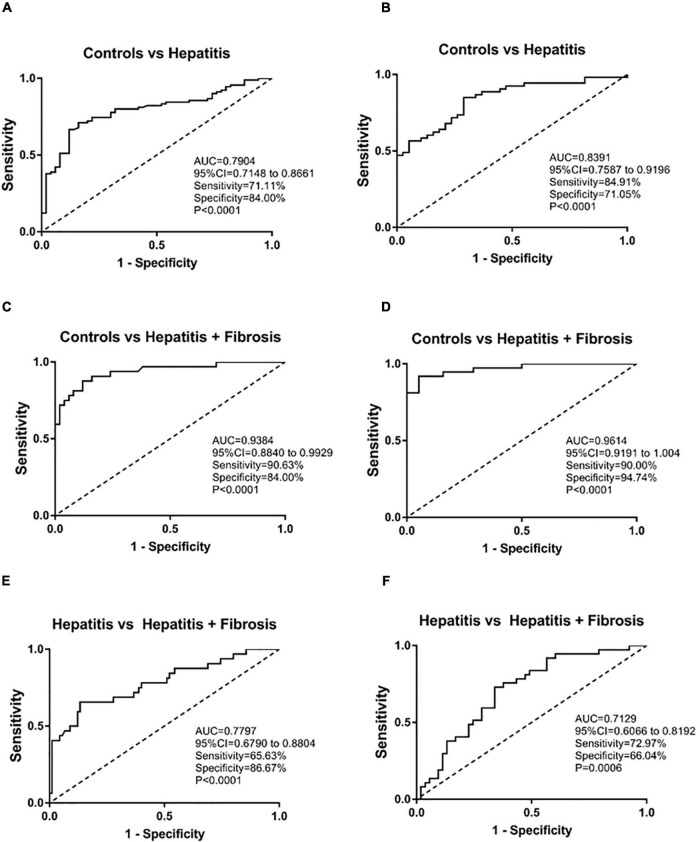
ROC analysis of sTim-3 to determine its diagnostic value for patients with hepatitis and hepatitis with fibrosis. **(A)** ROC curve of sTim-3 in healthy controls and patients with hepatitis (the first batch of included samples). **(B)** ROC curve of sTim-3 in healthy controls and patients with hepatitis (the second batch of included samples). **(C)** ROC curve of sTim-3 in healthy controls and patients with hepatitis and fibrosis (the first batch of included samples). **(D)** ROC curve of sTim-3 in healthy controls and patients with hepatitis and fibrosis (the second batch of included samples). **(E)** ROC curve of sTim-3 in patients with hepatitis and hepatitis with fibrosis (the first batch of included samples). **(F)** ROC curve of sTim-3 in patients with hepatitis and hepatitis with fibrosis (the second batch of included samples).

We divided the samples into two batches according to the time of inclusion of samples. The first batch was included from 2020.1 to 2020.6 and the second batch was included from 2020.7 to 2020.12. The second batch is used as the validation set. The ROC curve was used in evaluating the role of sTim-3 in distinguishing hepatitis from hepatitis with fibrosis. As shown in Figures 4A,C,E sTim-3 can be effectively used in distinguishing healthy controls and patients with hepatitis (AUC (area under the curve), 0.7904; sensitivity, 71.11%; specificity, 84.00%), controls and patients with hepatitis and fibrosis (AUC, 0.9384; sensitivity, 90.63%; specificity, 84.00%), and hepatitis and hepatitis with fibrosis as well (AUC, 0.7797; sensitivity, 65.63%; specificity, 86.67%). In the validation set, as shown in Figures 4B,D,F, sTim-3 can also be effectively used in distinguishing healthy controls and patients with hepatitis (AUC, 0.8391; sensitivity, 84.91%; specificity, 71.05%), controls and patients with hepatitis and fibrosis (AUC, 0.9614; sensitivity, 90.00%; specificity, 94.74%), and hepatitis and hepatitis with fibrosis as well (AUC, 0.7129; sensitivity, 72.97%; specificity, 66.04%).

As shown in Table 3, we compared the positive detection rates of sTim-3 and HA in patients with hepatitis and hepatitis with fibrosis. The cut off for sTim-3 was determined by the Youden index in Figure 4E, where the cut off value was 19.45 ng/mL. At this cut off value, and positivity rates of 21.68% and 66.13%, respectively, sTim-3 can effectively distinguishing between hepatitis and hepatitis with fibrosis. In addition, combined detection of HA and sTim-3 can improve the positive detection rate of hepatitis with fibrosis up to 77.43%.

**TABLE 3:** Comparison of the positive rate of sTim-3 and HA to distinguish hepatitis from hepatitis with liver fibrosis.

		Positive rate		
	Cut off	Controls	Hepatitis	Hepatitis + fibrosis
sTim-3	19.45 ng/mL	0.00%	21.68%	66.13%
HA	110 ng/mL	0.00%	4.90%	35.48%
sTim-3 or HA		0.00%	25.87%	77.42%

*The cut off for sTim-3 was determined by the Youden index of Figure 4E, and HA concentration greater than 110 ng/mL was considered positive (commonly used in clinical practice).*

## Discussion

We studied the role of sTim-3 in HBV, HCV, and HEV infections. Our results suggest that the sTim-3 concentrations in patients with hepatitis were significantly higher than those in the control group and were closely related to ALT and AST levels. sTim-3 is known to be related to the progression of infectious diseases, such as tuberculosis (18), HBV (10), and HIV (19). Serum sTim-3 may also be related to HCV and HEV infections and disease progression. Our study provides new information on the clinical diagnostic and surveillance value of sTim-3 in infectious diseases.

It is well known that Th cells play a key role in the regulation of the dynamic balance of the body’s immune response and an important regulatory role in HBV infection. Th cells secrete a variety of cytokines, increase the activity of CD8 + T cells, and promote cellular immune responses (20) improving the body’s ability to clear viruses. However, they also aggravate liver cell damage. ALT and AST are liver function parameters, and their levels increase in a patient’s serum upon cellular damage (10, 21). Increase in bilirubin level during viral hepatitis is related to liver cell damage and the degree of hepatic necrotizing inflammation (22), while reduction in ALB level is also related to the severity of liver synthetic function damage (10). The present study showed that sTim-3 levels in patients infected with HBV, HCV, or HEV were positively correlated with serum ALT, AST, Dbil, and Tbil levels, and sTim-3 levels were negatively correlated with ALB. The results indicate that sTim-3 can reflect the degree of liver cell damage in patients infected with HBV, HCV, or HEV to a certain extent and help in monitoring and evaluation of disease development.

This study showed that the sTim-3 concentrations in patients infected with HBV, HCV, and HEV were significantly higher than those in the healthy controls. The possible reason could be the accumulation of tissue-related memory Treg cells expressing high levels of Tim-3 in liver during the inflammatory stage of viral hepatitis infection (23). In addition, Tim-3 is mainly present on the surface of Th1 cells. When the body is invaded by viruses, Th1 levels increase during the activation of T cell immunity. Tim-3 expression on Th1 cells increases, which inhibits their excessive proliferation, and regulates the balance of Th1/Th2 (24), and exerts a negative immunomodulatory effect. Tim-3 expression also increases in depleted T cells and natural killer cells in patients infected with HBV/HCV (12, 13, 25, 26). Tim-3 mainly exists in three forms: full-length, spliced, and detached. Membrane bound Tim-3 can be cleaved from cell surfaces by metalloproteinases ADAM10 and ADAM17 to produce sTim-3 (27). sTim-3 can be a result of shedding from the cell surfaces after metalloprotease activity, and sTim-3 level may sequentially reflect membrane Tim-3 level. Therefore, sTim-3 levels in patients with three types of viral hepatitis infection were higher than those in the normal control group. The level of sTim-3 in patients infected with HEV was higher than that in patients infected with HBV, possibly because hepatitis caused by HEV is a systemic disease. Recently, studies have systematically explained the many extrahepatic manifestations of HEV infection, which are mainly related to diseases such as the kidney system, autoimmune system, and pancreatitis (28). Moreover, studies have shown that sTim-3 plays an important immunoregulatory role in kidney disease (17, 29), pancreatitis (30), and autoimmune diseases (31), resulting in its high serum levels. Similarly, sTim-3 levels were higher in patients infected with HEV as well.

sTim-3 concentration is elevated in patients infected with HBV and gradually increases in the serum of patients with liver cirrhosis and liver cancer (10). These findings are consistent with our research. In addition to HBV infection, our study reported for the first time that sTim-3 increased in patients infected with HCV and HEV as well and further increased in patients with hepatitis along with liver fibrosis. sTim-3 likely participates in pathogenesis by regulating the balance between Th1 and Th2. Liver fibrosis is a manifestation of tissue damage and inflammation, and viral hepatitis is one of its causes (32). Especially after hepatitis virus infection, without intervention treatment, liver fibrosis develops easily. Tim-3 may promote the progression of liver fibrosis by regulating the secretion of cytokines (TNF-α, IL-10, and IFN-γ) by immune cells, thereby regulating the progression of liver disease (33). Our data showed that Dbil and ALB in the serum of patients infected with HBV or HCV and hepatitis or hepatitis with fibrosis showed significant differences, but there was no difference among patients infected with HEV. Hence, sTim-3 has the advantage of distinguishing HEV-infected hepatitis and hepatitis with fibrosis. The dysfunction of monocytes is an important reason for the development of liver cirrhosis, and studies have confirmed that Tim-3 plays an important role in the expression of monocytes (26). High expression of Tim-3 may limit the activation of monocytes, thereby inhibiting cytokine production, which further promotes liver fibrosis progression. In addition, studies have shown that in patients with liver cirrhosis the CD^+^ T cell phenotype is altered, and high expression of Tim-3 in the cell membrane is one of the characteristics (34). This may be the reason for higher sTim-3 level in patients with liver fibrosis than in patients with hepatitis. These results indicate that sTim-3 level is related to the progression of the disease (the degree of liver damage) after hepatitis virus infection and is a potential marker for monitoring the progression of the disease upon hepatitis virus infection.

The results of this study indicate that sTim-3 has a certain diagnostic value to detect the degree of liver fibrosis in patients with hepatitis. HA is a commonly used clinical indicator for diagnosing liver fibrosis. When we combined sTim-3 and HA to diagnose liver fibrosis, the positivity rate significantly increased. Faster and more accurate diagnosis tools combined with effective treatment of viral hepatitis will help prevent proliferation of the disease. HEV infection is usually asymptomatic but can cause acute liver damage and even liver failure (35). Therefore, understanding the pathogenesis of hepatitis virus is essential for the treatment of the disease. In animal models, Tim-3 pathway blockade combined with PD-1 and CTLA-4 blockade can increase the production of T cells, reduce the expression of Treg, and restore T cell mediated immune response (36). Blocking the Tim-3/galectin-9 pathway might restore the secretion of some cytokines (IL-2, TNF-α, and IFN-γ) and T cell proliferation (37). These finding provides novel insights into the treatment of viral infectious diseases.

This study has several limitations including the small number of patients in the subgroups of different clinical diseases, and the lack of functional studies concerning the role of sTim-3 in the CD8 + T -cell exhaustion in viral hepatitis infection. We found that the combined detection of sTim-3 and HA can improve the positive detection rate of patients with liver fibrosis, but it only demonstrated moderate performance for discriminating hepatitis from liver fibrosis. Therefore, further studies are required to verify and extend our findings.

## Conclusion

sTim-3 levels were significantly increased in patients infected with HBV, HCV, or HEV. The quantitative detection of sTim-3 levels can be a tool to distinguish viral hepatitis from hepatitis with fibrosis. The level of sTim-3 is related to disease progression upon hepatitis virus infection.

## Data Availability Statement

The original contributions presented in the study are included in the article/supplementary material, further inquiries can be directed to the corresponding author/s.

## Ethics Statement

This manuscript is part of a study which was approved by the Wuxi No.5 People’s Hospital (approval no. 2020-023-1) and our study obtained informed consent from all participants. The patients/participants provided their written informed consent to participate in this study.

## Author Contributions

LC, BH, YD, and HP contributed to conception and design of the study. LC organized the database, performed the statistical analysis, and wrote the first draft of the manuscript. XZ, YQ, LC, SZ, YW, XY, and CL wrote sections of the manuscript. All contributed to manuscript revision, read, and approved the submitted authors version.

## Conflict of Interest

The authors declare that the research was conducted in the absence of any commercial or financial relationships that could be construed as a potential conflict of interest.

## Publisher’s Note

All claims expressed in this article are solely those of the authors and do not necessarily represent those of their affiliated organizations, or those of the publisher, the editors and the reviewers. Any product that may be evaluated in this article, or claim that may be made by its manufacturer, is not guaranteed or endorsed by the publisher.
